# *In vitro* Evaluation of Mannosylated Paromomycin-Loaded Solid Lipid Nanoparticles on Acute Toxoplasmosis

**DOI:** 10.3389/fcimb.2020.00033

**Published:** 2020-02-13

**Authors:** Mojdeh Khosravi, Hanieh Mohammad Rahimi, Delaram Doroud, Elnaz Sadat Mirsamadi, Hamed Mirjalali, Mohammad Reza Zali

**Affiliations:** ^1^Foodborne and Waterborne Diseases Research Center, Research Institute for Gastroenterology and Liver Diseases, Shahid Beheshti University of Medical Sciences, Tehran, Iran; ^2^Regulatory Department, Production and Research Complex, Pasteur Institute of Iran, Tehran, Iran; ^3^Department of Microbiology, Faculty of Medicine, Tehran Medical Sciences, Islamic Azad University, Tehran, Iran; ^4^Gastroenterology and Liver Diseases Research Center, Research Institute for Gastroenterology and Liver Diseases, Shahid Beheshti University of Medical Sciences, Tehran, Iran

**Keywords:** *Toxoplasma gondii*, acute toxoplasmosis, paromomycin, mannosylation, solid lipid nanoparticles

## Abstract

*Toxoplasma gondii* is a zoonotic intracellular protozoan with worldwide distribution. Acute and severe toxoplasmosis are commonly reported in patients who suffer from acquired/congenital immune deficiency. This study aimed to synthesize mannosylated paromomycin-loaded solid lipid nanoparticles (PM-SLN-M) and to evaluate them on acute toxoplasmosis. SLN was synthesized and then loaded by 7 mg/mL paromomycin sodium. Mannose coating was performed, and after washing, the size, zeta potential, and loading percentage were calculated. To evaluate the cell toxicity, an MTT assay was performed on Vero cells by different concentrations (log 10^−1^) of SLN, PM-SLN-M, and PM-SLN. In addition, the anti-*Toxoplasma* effects were also evaluated using trypan-blue staining and scanning electron microscopy (SEM). An MTT assay was also employed to evaluate the effects of PM and PM-SLN-M on intracellular *Toxoplasma*. A 6-month stability test of PM-SLN and PM-SLN-M represented that the characteristics all remained constant. The cell viability assay demonstrated that PM-SLN-M had lower cell toxicity (<20%) compared to PM-SLN (<30%) and PM (<40%). Statistical analysis showed that PM-SLN-M significantly killed ~97.555 ± 0.629 (95% CI: 91.901 to 103.209; *P* < 0.05) of *T. gondii* tachyzoites. More than 50% of *Toxoplasma-*infected Vero cells remained viable in concentrations more than 0.07 μg/mL and 7 μg/mL of PM and PM-SLN-M, respectively. SEM analysis showed that *T. gondii* tachyzoites were changed in both size and morphology facing with PM-SLN-M. Our findings indicated that synthesized PM-SLN-M had anti-*Toxoplasma* activity without significant host cell toxicity at the highest concentration. Our study demonstrated that PM was able to kill intracellular *Toxoplasma* in lower concentration in comparison to PM-SLN-M, although PM-SLN-M showed lower cytotoxic effects on Vero cells.

## Introduction

*Toxoplasma gondii* is a zoonotic obligate intracellular protozoan parasite that infects a broad range of warm-blooded animals from birds to humans. Global estimation shows 30–50% of the world's population are serology positive for toxoplasmosis (Robert-Gangneux and Darde, 2012; Flegr et al., 2014; Aguirre et al., 2019). The prevalence rates 0–100% were reported for toxoplasmosis regarding the methods of detection and geographical regions (Robert-Gangneux and Darde, 2012; Liu et al., 2015; Rostami et al., 2018).

*T. gondii* has three main stages during its life cycle, including the tachyzoite, tissue cyst (bradyzoite), and oocyst phases, of which tachyzoite is responsible for cell invasion, and acute phase of the disease (Kato, 2018). Although ingestion of mature oocyst via food and water is the main route of infection (Hill and Dubey, 2016), consumption of raw meat containing cysts, needle injection (particularly in research laboratory), congenital infection, blood transfusion (particularly white blood cells), and organ transplantation from an infected person are the other potential routes of transmission (Montoya and Liesenfeld, 2004).

Pathogenesis of *T. gondii* may differ based on its genotypes/strains; however, it seems that toxoplasmosis is asymptomatic in more than 80% of immunocompetent subjects (Montoya and Liesenfeld, 2004). Toxoplasmosis in immunocompromised patients who do not properly respond to the infection is the main challenge. Accordingly, acute infection due to toxoplasmosis ranks high on the list of fatal diseases in HIV/AIDS patients (Hill and Dubey, 2002; Ahmadpour et al., 2019). Indeed, acute and severe toxoplasmosis are commonly reported from transplant recipients (Martina et al., 2011; Paccoud et al., 2019; Schmidt-Hieber et al., 2019), cancer patients (Fox et al., 2017; Bajnok et al., 2019; Melchor and Ewald, 2019), and patients who suffer from acquired/congenital immunodeficiency (Vijaykumar et al., 2018; McDermott et al., 2019). Taken together with the complications due to acute toxoplasmosis in immunocompromised patients, more recently, the potential role of activated toxoplasmosis in response to the immunomodulatory drugs during inflammatory bowel diseases (IBD) and its pathogenesis was suggested (Mirjalali et al., 2019). Therefore, prescription of a standard drug regimen with low side effects is an important issue during acute toxoplasmosis.

Pyrimethamine and sulfadiazine are standard drugs of choice in the case of acute toxoplasmosis. However, because of their adverse and side effects, the need for a new treatment plan has been highlighted (Martins-Duarte et al., 2006; Alday and Doggett, 2017).

Paromomycin (aminosidine; PM) is an aminoglycoside–aminocyclitol antibiotic with a broad-spectrum toxicity against bacteria, as well as some parasites such as *Giardia, Leishmania*, and *Entamoeba histolytica* (Kappagoda et al., 2011). This drug faces many challenges, such as quick renal excretion and short half-life in the blood circulation. Indeed, this drug is hydrophilic and has a high molecular weight that makes its uptake difficult (Ghadiri et al., 2012; Afzal et al., 2019). However, PM can be a good option for treatment of parasitic disease due to its safety, low cost, and short course therapy.

Over the years, drug delivery systems have been experienced as a way to enhance the effectiveness of some drugs such as PM (Ghadiri et al., 2012; Gaspar et al., 2015). So far, liposomes have been used as a drug delivery system in many studies where the results indicated improved penetration properties and therapeutic effects of PM (Gaspar et al., 2015; Heidari-Kharaji et al., 2016a). The current study aimed to evaluate the cell toxicity and anti-*Toxoplasma* potency of PM-SLN-M, *in vitro*.

## Methods

### Preparation of Uncoated SLNs Loaded by PM (PM-SLN)

SLN was prepared by solvent injection method according to the procedure reported by Schubert and Muller-Goymann with slight modifications (Schubert and Muller-Goymann, 2003). Briefly, tristearin (1% w/v) and soya lecithin (PC; 0.3% w/v) were dissolved in a 10-mL mixture of acetone and ethanol (1:1 v/v) at 70°C. Then, stearyl amine (SA) was added into the mixture with the ratio of 10 mol % of soya lecithin. Temperature was kept at 70°C during the process. The melt was rapidly injected through a syringe at a flow rate of 5 mL/min into a stirred aqueous phase (with 0.2% w/v Tween 80) containing PM (0.1% w/v) maintained at the same temperature. The suspension was stirred at 4,000 rpm for 1 h, sonicated for 5 min, and then was filtered through membrane filter (0.45 μm) to remove any excess lipid. After this process, the PM-SLNs were ready for coating with mannose.

### Preparation of Mannosylated SLNs Loaded by PM (PM-SLN-M)

**Figure 1:**
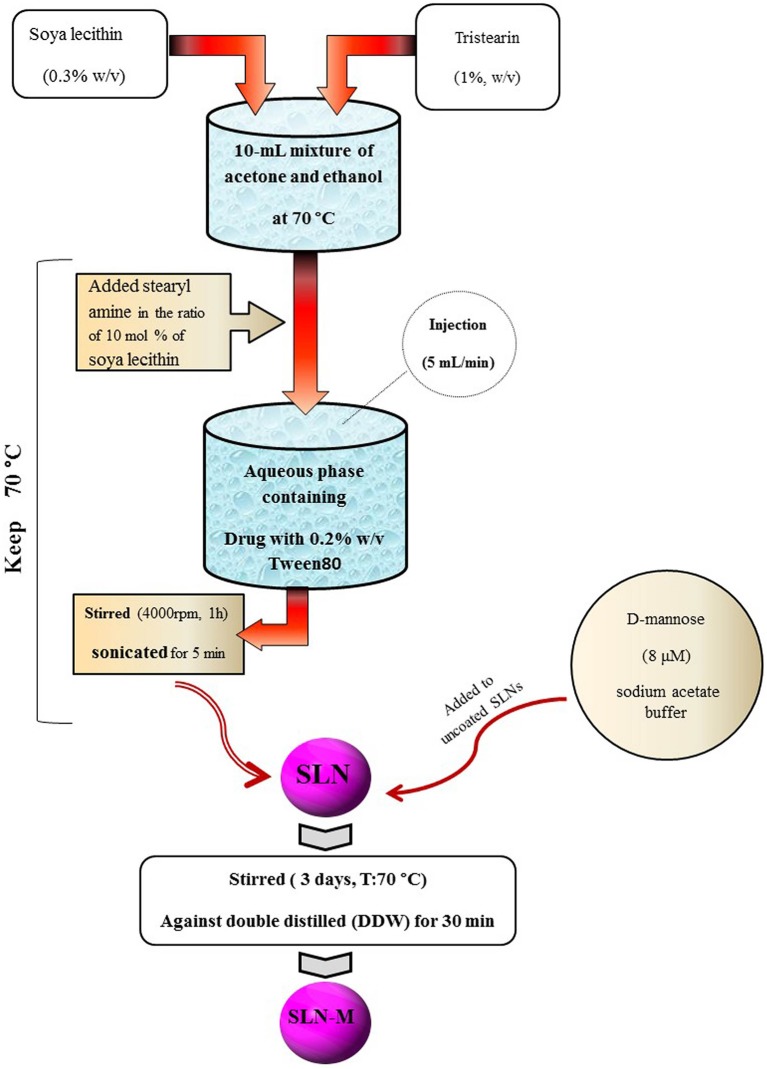
Schematic protocol describing the processing of PM-SLN-M synthesis.

Mannose coating was carried out in accordance with the previously reported method (Kumar et al., 2006). D-mannose (8 μM) was dissolved in sodium acetate buffer (pH 4.0; 0.1 M) and was added to uncoated SLNs. The mixture was continuously stirred using magnetic stirrer at room temperature for 3 days. Mannosylated nanoparticles were then subjected to extensive dialysis (dialysis bag; MWCO 12–14 kDa, Himedia, India) against double distilled water (DDW) for 30 min to remove uncoated mannose and other impurities (Ghadiri et al., 2011). Figure 1 schematically overviews the preparation of PM-SLN-M (Figure 1).

### Drug Content Determination

Photon correlation spectroscopy was employed to measure the size, pumulates mean size (z-potential), and the polydispersity index (PDI) of the particles (Zetasizer Nano Series, Malvern, UK) at 25°C. The amount of PM in supernatant was determined by high performance liquid chromatography (HPLC) as mentioned elsewhere (Pick et al., 1997).

### Cell Culture

In the present study, African green monkey kidney cells (Vero) were used for *in vitro* assay. Accordingly, Vero cells were grown in Dulbecco's Modified Eagle Medium (DMEM; Gibco BRL, USA) supplemented with 10% fetal bovine serum (FBS; Gibco BRL) and 1% penicillin/streptomycin (Gibco BRL) at pH 7.2, 37°C, and 5% CO_2_.

### Parasite

Tachyzoites of *T. gondii* (RH strain), which had been passaged in BALB/c mice (8–10-week-old, 20–25 g weight) using intraperitoneal (IP) injection, were washed with sterile phosphate-buffered saline (PBS; pH 7.4). After counting by hemocytometer slide, 1.5 × 10^6^ tachyzoites per mL was incubated with ~10^5^ Vero cells [multiplicity of infection (MOI) = 10], cultivated in DMEM supplemented with 10% FBS and 1% penicillin/streptomycin for mass cultivation. Every 3–5 days, the cells were checked to investigate the *Toxoplasma* growth. After maximum cell rupture and releasing tachyzoites, the parasites were harvested for further analyses.

### Cell Cytotoxicity Assay

To evaluate the toxicity effects of the synthesized nanocomponents, the viability rate of Vero cell against six log^−10^ concentrations (7 mg/mL−70 ng/mL) of SLN, PM-SLN, and PM-SLN-M was evaluated using MTT assay. Briefly, 2.38 × 10^5^ Vero cells in the exponential growth stage were seeded in a 96-well cell plate containing DMEM supplemented with 10% FBS for 48 h without any antibiotics. Then, the cells were treated with SLN, PM-SLN, PM-SLN-M, and PM sodium (log^−10^ from 7 mg/mL to 70 ng/mL). A well of Vero cells without treatment was considered as negative control. After 48 h incubation, the culture supernatant was removed and 15% (v/v) of MTT solution (5 mg/mL) was directly added to the wells, and the plate was incubated for 4 h at 37°C in 5% CO_2._ Formazan extraction was performed by incubation with Me_2_SO (150 μL/well) at 37°C for 10 min, and the absorbance of the plates at 570 nm was read with ELISA reader (LX800; Biotec, Winooski, VA, USA).

Toxicity effects of the defined concentrations were calculated according to the following equation. The minimum concentrations that inhibited an average of 50% of the parasites and host cells were considered as inhibitory concentration (IC50) and cytotoxic concentration (CC50), respectively.

Viable microorganisms % = [(AT – AB) / (AC – AB)] × 100

Nonviable microorganisms % = 100 – Viable microorganisms %

AT is the OD of treated well, AC is the OD of negative control, and AB is the OD of the blank well. A well of Vero cells without treatment and a well with only the media were considered as negative control and blank, respectively. Mean ± SD of the experiments was reported for each sample.

### Parasite Toxicity Assay

To study the anti-*Toxoplasma* activity of PM-SLN-M, six concentrations of PM (as positive control) and PM-SLN-M (log^−10^ from 7 mg/mL to 70 ng/mL) were added to 10^5^ parasites per well of a 96-well cell culture plate containing DMEM supplemented with 10% FBS without antibiotic. After 2 h incubation in 37°C and 5% CO_2_, the number of alive *Toxoplasma* tachyzoites was counted using vital staining (trypan-blue) and hemocytometer slide.

Viability and Non-viability data was calculated using the following equation:

Nonviable microorganisms % = (PT / NT) × 100

Viable microorganisms % = 100 – Nonviable microorganisms %

^*^PT is the number of parasite in each test well; NT is the number of parasite in a well without treatments. Mean ± SD of the experiments was reported for each sample.

### Ratio

To determine the best concentration of PM-SLN-M and compare the results to PM, the anti-*Toxoplasma* effects and the cell viability percentage were calculated using the following formula. The concentration closer to 1 was considered as the best dosage.

$$\begin{array}{l} Ratio=\frac{\text{Killed }Toxoplasma\;\left(\%\right)}{\text{Viable Vero cells }\left(\text{\%}\right)} \end{array}$$

### Intracellular Anti-*Toxoplasma* Activity

In order to investigate the effects of PM-SLN-M on the intracellular *Toxoplamsa*, 10^5^ Vero cells were seeded in a 96-well cell culture plate containing DMEM supplemented with 10% FBS without antibiotics. After ~70–80% confluency, 10^5^ tachyzoites of *Toxoplasma* (MOI = 1) were inoculated in each well, and the plate was incubated at 37°C and 5% CO_2_ for 24 h. Then, the wells were checked by inverted light microscope to confirm the maximum cellular invasion by the parasites. Afterwards, the culture supernatant was removed, and wells were washed twice by sterile PBS to remove cell debris, dead cells, and extracellular tachyzoite. The cells were then treated with PM-SLN-M and PM sodium (log^−10^ from 7 mg/mL to 70 ng/mL), together with DMEM supplemented with 10% FBS for 48 h. Finally, the culture supernatant was removed and MTT assay was performed.

### Scanning Electron Microscopy (SEM)

To evaluate the anti-*Toxoplasma* effects of PM-SLN-M formulation, after 2-h exposure of 10^5^ *Toxoplasma* tachyzoites with the nanodrug, tachyzoites were attached on a slide, fixed with 2% paraformaldehyde and 2.5% glutaraldehyde in 0.1 M sodium cacodylate buffer (pH 7.4) and washed in cacodylate buffer. Then, the slide was post-fixed for 2–4 h using 1–2% osmium tetroxide in 0.1 M phosphate buffer (pH 7.2) at room temperature and dehydrated in graded ethanol dilutions (70, 80, 90, and 100%). Cells were dried using critical point method, mounted on stubs, coated with gold (20–30 nm), and then observed using SEM.

### Statistical Analysis

One-sample *t*-test incorporated in GraphPad Prism software (version 8.0.2) was employed to calculate mean ± SD, confidence interval, and statistical correlation between concentration and components. *P* < 0.05 was considered as statistically significant.

## Results

### PM-SLN-M Characterization and Properties

**Figure 2:**
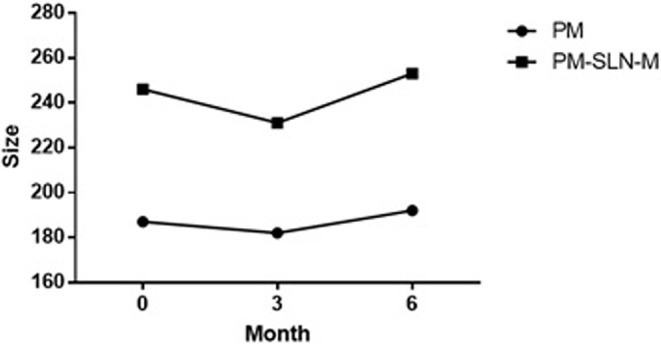
The sustainability results of PM-SLN-M during 6-month evaluation.

Specific properties of prepared PM-SLN-M such as size, polydispersity index, percentage of PM loading, and zeta potential of the particles are summarized in Table 1. The average size of the particles was 246 ± 32 nm, and the stability of the formulation (accelerated, 6 months), which was evaluated based on protocol ISCH-Q6 and stored in the refrigerator, showed good results (Figure 2). The release of drug from the PM-SLN-M formulation was slow and lasted for 24 h in the aqueous media. Indeed, the average entrapment efficiency of PM-SLN-M was 47–48%.

**Table 1:** Specific properties of synthesized nanoparticles.

**Formulation**	**Polydispersity index**	**Zeta potential (mV)**	**Size (nm)**
PM-SLN	0.26 ± 0.4	+31.3 ± 1.5	187 ± 14
PM-SLN-M	0.8 ± 0.02	+25.6 ± 1.7	246 ± 32

### Nanocomponent Toxicity Assay

**Figure 3:**
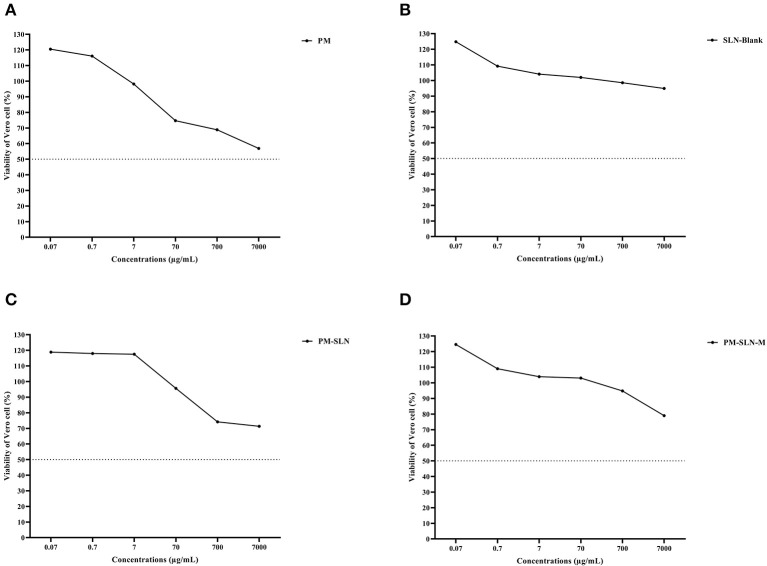
Cell toxicity assay of **(A)** PM, **(B)** SLN-BLANK, **(C)** PM-SLN, and **(D)** PM-SLN-M on Vero cell line.

Toxicity assay of nanoformulations showed that more than 78.96 ± 0.07% (95% CI: 78.325–79.595%) of Vero cells remained viable at the highest concentration (7 mg/mL) of PM-SLN-M (CC50 > 7 mg/mL) while almost 56.85 ± 0.255% (95% CI: 54.563–59.137%) of the cells dead using the same concentration of PM (*P* < 0.05; CC50 > 7 mg/mL). Comparison of cytotoxicity effects of PM-SLN with PM-SLN-M showed that mannosylation reduced the cell toxicity of the nanodrug from ~30 to 20% of Vero cells at the highest concentration. Indeed, toxicity effects of only SLN was evaluated that showed this component was not toxic for Vero cell, even at the highest concentration (Figure 3; Table 2).

**Table 2:** Vero cell viability against different concentrations of PM, PM-SLN, and PM-SLN-M.

**Concentrations (μg/mL)**	**PM**		**PM-SLN**		**PM-SLN-M**		***P* < 0.05**
	**Mean ± SD (%)**	**95% CI (%)**	**Mean ± SD (%)**	**95% CI (%)**	**Mean ± SD (%)**	**95% CI (%)**	
7,000	56.85 ± 0.255	54.563–59.137	71.345 ± 0.431	67.469–75.220	78.96 ± 0.07	78.325–79.595	^*^
700	68.84 ± 0.240	66.680–71	74.155 ± 0.176	72.566–75.743	94.815 ± 0.290	92.210.3–97.420	
70	74.655 ± 0.488	70.271–79.039	95.62 ± 0.608	90.156–101.083	103.06 ± 0.085	102.298–103.822	
7	98.165 ± 0.219	96.195–100.134	117.49 ± 0.692	111.263–123.716	103.9 ± 0.297	101.232–106.568	
0.7	116.055 ± 0.078	115.356–116.754	117.93 ± 0.678	111.831–124.028	109.03 ± 0.255	106.743–111.317	
0.07	120.485 ± 0.728	113.941–127.029	118.845 ± 0.219	116.875–120.814	124.57 ± 0.608	119.106–130.34	

* *Statistically significant*.

### Anti-*Toxoplasma* Effects of PM-SLN-M

**Figure 4:**
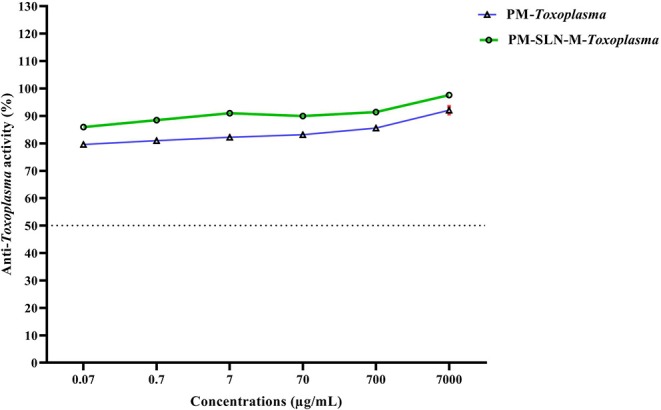
Comparison of anti-*Toxoplasma* activity of PM and PM-SLN-M according to different concentrations.

The results of vital staining showed that at the highest concentration, PM-SLN-M was able to kill 97.555 ± 0.629% (95% CI: 91.901–103.209%) of tachyzoites, in comparison to PM (92.065 ± 1.322% [95% CI: 80.185–103.945%]; IC50 > 0.07 μg/mL). In addition, at the lowest concentration (0.07 μg/mL), PM-SLN-M killed 85.895 ± 0.148% (95% CI: 84.561–87.229%), while PM was toxic for 79.55 ± 0.636% (95% CI: 73.832–85.268%) of *Toxoplasma* tachyzoites (IC50 > 0.07 μg/mL) (Figure 4; Table 3).

**Table 3:** Anti-*Toxoplasma* activity of different concentrations of PM and PM-SLN-M and the ratio value of anti-parasite activity per Vero cell viability.

**Concentrations (μg/mL)**	**PM**			**PM-SLN-M**			***P* < 0.05**
	**Mean ± SD (%)**	**95% CI (%)**	**Ratio**	**Mean ± SD (%)**	**95% CI (%)**	**Ratio**	
7,000	92.065 ± 1.322	80.185–103.945	1.62	97.555 ± 0.629	91.901–103.209	1.23	^**^
700	85.575 ± 0.615	80.048–91.102	1.24	91.365 ± 0.516	86.727–96.003	0.963^*^	
70	83.135 ± 0.049	82.690–83.580	1.11^*^	89.95 ± 0.071	89.315–90.585	0.872	
7	82.23 ± 0.325	79.308–85.152	0.837	90.985 ± 0.290	88.380–93.59	0.875	
0.7	80.945 ± 0.078	80.246–81.644	0.697	88.45 ± 0.636	83.732–94.168	0.811	
0.07	79.55 ± 0.636	73.832–85.268	0.66	85.895 ± 0.148	84.561–87.229	0.689	

* The ratios closer to 1 show the best concentrations of drugs with highest anti-Toxoplasma activity and lowest Vero toxicity.

** *Statistically significant*.

### Anti-intracellular *Toxoplasma* Effects of PM-SLN-M

**Figure 5:**
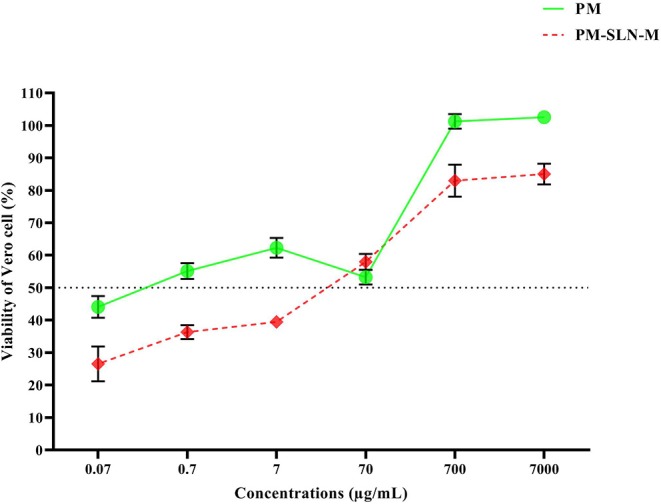
The effects of different concentrations of PM and PM-SLN-M on intracellular *Toxoplasma*.

The results represented that at least 50% of the infected Vero cells treated by the concentration higher than 7 μg/mL of PM-SLN-M remained viable (IC 50 > 7 μg/mL). Moreover, PM was able to kill *Toxoplasma* in the infected Vero cells by concentrations more than 0.07 μg/mL (IC 50 > 0.07 μg/mL; Figure 5; Table 4).

**Table 4:** Anti-intracellular *Toxoplasma* activity of different concentrations of PM and PM-SLN-M.

**Concentrations (μg/mL)**	**PM**		**PM-SLN-M**		***P* < 0.05**
	**Mean ± SD (%)**	**95% CI (%)**	**Mean ± SD (%)**	**95% CI (%)**	
7,000	102.506 ± 1.728	98.214–106.799	84.996 ± 3.204	77.038–92.955	^*^
700	101.203 ± 2.250	95.614–106.792	82.95 ± 4.927	70.71–95.19	
70	53.223 ± 2.273	47.577–58.869	57.93 ± 2.437	51.8840–63.989	
7	62.286 ± 3.019	54.786–69.787	39.446 ± 1.37	36.042–42.851	
0.7	55.106 ± 2.465	48.982–61.231	36.336 ± 2.139	31.024–41.649	
0.07	44.096 ± 3.349	35.776–52.417	26.506 ± 5.417	13.051–39.962	

* *Statistically significant*.

### Ratio Analysis

**Figure 6:**
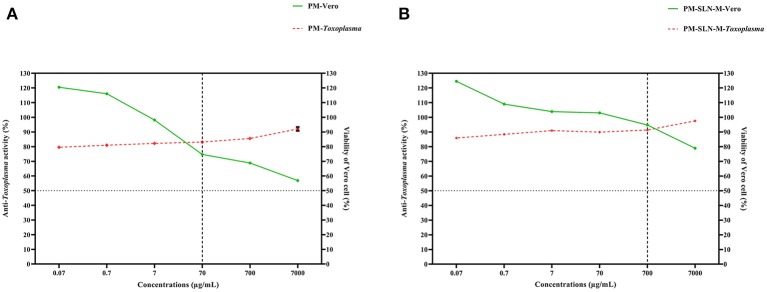
Ratio analysis of **(A)** PM and **(B)** PM-SLN-M shows that PM-SLN-M at the higher concentration than that in PM (700 vs. 70 μg/mL) revealed highest and lowest anti-*Toxoplasma* activity and Vero cell toxicity, respectively.

The comparison of the efficacy to the safety of PM-SLN-M and PM, ratio analysis was performed that the results showed PM-SLN-M at the concentration 700 μg/mL and ratio 0.963 had the highest and lowest toxicity for *Toxoplasma* tachyzoites and Vero cells, respectively. Indeed, PM showed the highest and lowest toxicity for *Toxoplasma* tachyzoites and Vero cells, respectively, at the concentration 70 μg/mL with ratio 1.11 (Figure 6).

### Scanning Electron Microscopy

**Figure 7:**
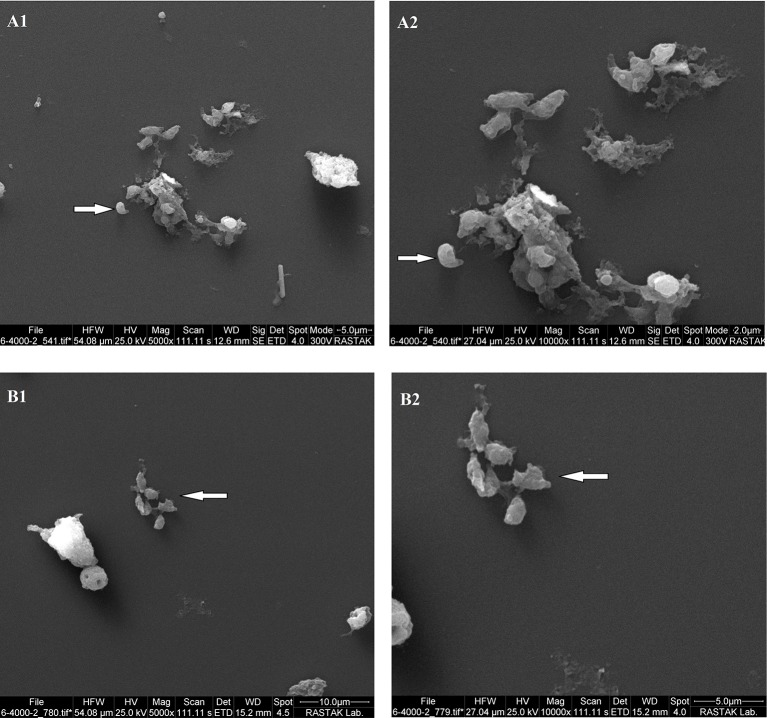
The results of SEM analysis reveals changes in size and morphology of *Toxoplasma* tachyzoites in two magnifications. **(A1,A2)** show untreated tachyzoites, and **(B1,B2)** show PM-SLN-M treated tachyzoites.

Morphological analysis of *T. gondii* tachyzoites treated with PM-SLN-M represented a non-crescent shape with abnormal surface, while tachyzoites in control group remained normal in shape and size with a smooth surface (Figure 7).

## Discussion

Acute severe toxoplasmosis in immunocompromised patients and women who are infected by *T. gondii* during the first pregnancy is the main challenge of physicians (Montoya and Liesenfeld, 2004). Although there are single or combined drug regimens facing with acute toxoplasmosis, most of the recommended drugs are not effective enough or may lead to side effects (Alday and Doggett, 2017). However, sulfadiazine-pyrimethamine, spiramycin, clindamycin, and atovaquone are known the drugs of choice, despite being of different efficacy and probable side effects (Alday and Doggett, 2017; Montazeri et al., 2017). It was claimed that the combination of sulfadiazine and pyrimethamine, recommended drugs for acute toxoplasmosis, may lead to immunosuppression and anemia due to clampdown of bone marrow production despite of its good efficacy (Crespo et al., 2000; Faucher et al., 2011). Indeed, although parasitocidal effects of atovaquone on protozoan parasites have been demonstrated, the need for high dosage due to low bioavailability is the main challenge in oral prescription of this drug (Pentewar et al., 2015; Darade et al., 2018).

Notably, different nanoformulations and nanomaterials have been used in practice and the results are promising. In addition, it was strongly claimed that utilizing nanomaterials in either combination or formulation of the conventional drugs not only decreases the probable side effects, but also increases their efficiency (Barabadi et al., 2019).

Although many nanoformulations of available drugs were tested either *in vivo* or *in vitro*, there is no data representing efficiency of PM and its nanoformulations on toxoplasmosis. Gaafar et al. (2014) worked on chitosan and silver nanoparticles either singly or in a component against *Toxoplasma* and reported significant reduction in the parasite count in the infected mice. Anti-*Toxoplasma* activity of different molecular weights and concentrations of chitosan was then evaluated by Teimouri et al. (2018), who reported significant anti-*Toxoplasma* toxicity of all molecular weights and concentrations. Azami et al. (2018a) assessed nanoemulsion of atovaquon for treatment of both chronic and acute toxoplasmosis and revealed that this component was toxic for both tachyzoites and brain cysts of *Toxoplasma*. At the same time, Azami et al. (2018b) evaluated curcumin nanoemulsion (CR-NE) on both chronic and acute toxoplasmosis and demonstrated that CR-NE can be considered a promising compound for the treatment of toxoplasmosis. However, in these studies, host cell toxicity was not assessed on the cell lines to reveal a ratio of anti-*Toxoplasma* effect to host cell toxicity. In the current study, PM-SLN-M showed highest anti-*Toxoplasma* activity and lowest host cell toxicity at the concentration 700 μg/mL, in comparison to PM, which showed highest anti-*Toxoplasma* activity and lowest host cell toxicity at the concentration 70 μg/mL. This finding suggests that SLN mannosylation not only increases anti-*Toxoplasma* activity, but also decrease host cell toxicity of PM.

Our results confirmed the previous studies showing non-toxicity of SLN-PM either *in vitro* or *in vitro*. PM is a broad-spectrum, well-known antibiotic whose bactericidal and parasiticidal properties are shown satisfactory. Although the hydrophilic structure of this drug makes penetration of PM to the target cells difficult, there are a number of studies that reported satisfactory outcomes that resulted from the nanoformulation of PM. Up to now, nanoformulation of PM was mostly practiced on different types of leishmaniasis. Heidari-Kharaji et al. (2016a,b) showed that PM-SLN was a non-toxic compound that increased the anti-*Leishmania* toxicity of PM.

Notably, short half-life and specific drug delivery of PM are the main challenges during clinical practices. It was demonstrated that SLN carriers can increase the half-life of PM through controlling the drug release. Ghadiri et al. (2012) examined entrapment efficiency and release profile of PM-loaded SLN using statistical modeling and showed that SLN led to gradually efficient prolonged release of PM. Interestingly, in immunocompromised patients, chronic cysts of *Toxoplasma* may rupture due to insufficient immune responses; thus, activated tachyzoites can lead to lethal complications. Therefore, since SLN controls efficient prolonged release of PM, it seems PM-SLN can remain effective for a long time.

Additionally, macrophages are the first line of defense during acute toxoplasmosis (Kato, 2018; Lima and Lodoen, 2019); therefore, increasing the drug uptake by macrophages is the most important challenge during treatment of acute toxoplasmosis. Mannose receptor, known as CD206, is mostly expressed by macrophages and dendritic cells. This receptor binds with mannose that usually covers outer surface of microbes and leads to scavenging pathogens (Azad et al., 2014). Notably, macrophages are the central host cells which are infected by intracellular pathogens such as *Leishmania* and *Toxoplasma*, and it was well-established that mannosylated PM-SLN not only increase the bioavailability of PM, but also mannosylation enhance uptaking PM by macrophages (Frenz et al., 2015). In the study conducted by Afzal et al. (2019), the maximum uptake was observed in macrophages that were treated by mannosylated thiolated chitosan-coated PM-loaded PLGA nanoparticles.

As a result, PM was able to kill intracellular *Toxoplasma* in lower concentration than PM-SLN-M. Nonetheless, regarding the lower cell toxicity of PM-SLN-M in comparison to PM, it seems that PM-SLN-M could be a better choice particularly in long-term therapy.

In summary, our findings demonstrate that although PM is usually used for therapy of giardiasis, leishmaniosis, and amoebiasis, nanoformulation of this drug may make it a useful drug for acute toxoplasmosis. Indeed, PM-SLN-M showed higher anti-*Toxoplasma* toxicity in comparison to conventional PM and mannosylation may increase up taking the drug by macrophages. Taken together, it seems that nanoformulation not only increases efficiency of available commercial drugs, but also decreases the cytotoxicity of them against host's cell.

## Data Availability Statement

All datasets generated for this study are included in the article/supplementary files.

## Ethics Statement

All procedures performed in this study were in accordance with the ethical standards (IR.SBMU.RIGLD.REC.1398.034) released by the Ethical Review Committee of the Research Institute for Gastroenterology and Liver Diseases, Shahid Beheshti University of Medical Sciences, Tehran, Iran.

## Author Contributions

HM designed the study. DD synthesized nanoparticles. MK, HMR, and HM contributed in performing the experiments and analyzing the generated data. MK, EM, and HM contributed in writing the manuscript. MZ supported and supervised the study.

### Conflict of Interest

The authors declare that the research was conducted in the absence of any commercial or financial relationships that could be construed as a potential conflict of interest.
